# Temporally modulated one-dimensional leaky-wave holograms

**DOI:** 10.1038/s41598-022-12432-w

**Published:** 2022-05-19

**Authors:** Amrollah Amini, Homayoon Oraizi

**Affiliations:** School of Electrical Engineering, Iran University of Science and Technology, Tehran, 1684613114 Iran

**Keywords:** Engineering, Electrical and electronic engineering

## Abstract

Spatio-temporally modulated impedance surfaces can be good candidates for generation of radiating waves with arbitrary eigenstates by breaking momentum and energy conservations. Here, we present a theoretical framework based on the holographic technique and generalized Floquet-wave expansion to analyze spatio-temporally modulated impedance surfaces. The holographic technique estimates the required impedance distribution to achieve the desired momentum. Injecting temporal modulation deviates the eigenvalues and changes the radiation frequency. Using the proposed analytical model, the eigenvalues can be calculated accurately in the presence of space and time modulations. Consequently, it is possible to predict the propagation mechanism of bounded and radiation states. It has been shown that, imposition of temporal modulation causes the Doppler-shift effect and nonreciprocal responses in the hologram. By plotting the antenna dispersion diagram, and observing the asymmetric displacement of dispersion curve due to temporal modulation, the system nonreciprocity can be verified. The beam scanning properties of these structures have also been investigated.

## Introduction

Recently, planar nonreciprocal devices without the use of bulky ferromagnetic materials have received much attention^1,2^ due to their subwavelength thicknesses and low-cost manufacturing processes. A suitable solution for implementing such devices is to exploit spatio-temporally modulated structures. This method can cover a wide range of frequencies from microwaves^3^ to optical^4,5^ regimes. The use of spatio-temporally modulated structures is not limited to electromagnetic devices, but can also be applied to manipulate acoustic waves^6^. A wide variety of potential applications can be envisaged for space-time structures, such as isolators^7^, frequency translators^8^, circulators^9^, and nonreciprocal phase shifters^10^. In the context of antenna engineering several works have also been proposed to realize nonreciprocal radiators^11–13^. A mixer-duplexer leaky-wave antenna is presented in the literature using temporally modulated microstrip lines^12^. An array of varactors incorporated in transmission lines was used to inject temporal modulation. A silicon-compatible leaky-wave antenna has been designed based on graphene impedance surfaces^13^. Nonreciprocity and Doppler-shift effects are obtained by applying temporal modulation through gating pads located adjacent to graphene surfaces. These structures can be used for bio-sensing, imaging, and inter-chip communication applications^13^.

An appropriate approach to implement spatio-temporally modulated structures is to use dynamic metasurfaces, which have several advantages such as ultra-thin thicknesses, low cost and high flexibility. Metasurfaces are mainly exploited in transmissive^14–20^, reflective^21–25^, or leaky-wave^26,27^ modes. The main drawback of microwave metareflectors and transmitarrays is their protruded feeds which make them unsuitable for low-profile integrated systems. This shortcoming can be resolved by using leaky-wave metasurfaces which are generally fed by embedded monopole structures. Owing to the above-mentioned attractive features, various methods have been recently proposed for the design and analysis of leaky-wave metasurfaces. The holographic technique^28–30^, aperture field estimation (AFE) method^31–33^, Floquet-wave expansion model^34,35^ and the method of moments (MoM)^36,37^ are among the proposed methods of designing metasurface-based leaky-wave antennas. The holographic and AFE techniques can be considered as synthesis methods for the estimation of arrangement of meta-atoms to achieve the desired wavefronts, whereas the MoM and Floquet-wave expansion are analysis frameworks for the determination of exact solutions of leaky-wave modes generated by the synthesized metasurfaces. Therefore, for a comprehensive study of leaky-wave metasurfaces, we need to combine the analysis and synthesis methods.

In this paper, the combination of holographic technique and generalized Floquet-wave expansion is proposed to analyze the temporally modulated one-dimensional leaky-wave metasurfaces. The scalar impedance boundary condition is used to model the proposed hologram. The generalized Floquet-wave expansion was first proposed by Cassedy^38^ to attain the mode characteristics of temporally modulated surface waveguides. In our work, this method is used for impedance surfaces to explain the radiation mode and the leakage mechanism in the presence of space-time modulation. Due to the nonreciprocity of temporal modulation, the antenna responds differently in the transmission and reception modes. Note that, in the transmission mode, the surface wave (slow wave) excited by the launcher leaks energy to the radiation mode (fast mode). In the reception mode, this process occurs in the reverse direction. That is, the radiation mode (fast wave) is converted to the surface slow wave. As an example, using the holographic technique, the surface impedance is designed to radiate in the direction of 30° at 18 GHz. The effect of temporal injection is studied and the radiation characteristics of antenna in the transmission and reception modes have been investigated.

## Spatio-temporally modulated impedance surfaces

**Figure 1:**
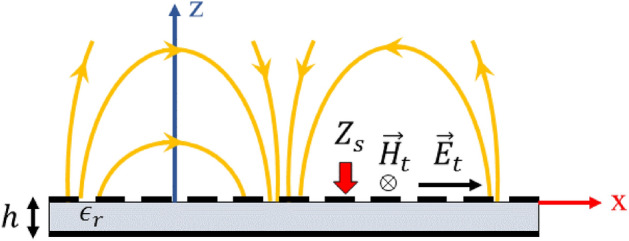
Surface impedance boundary condition implemented on a grounded dielectric slab. The propagation direction is assumed to be along the x-axis.

A convenient way to implement holographic metasurfaces in the microwave regime is to modulate impedance surfaces. For an impedance surface located at $z = 0$ (see Fig. 1), the tangential components of electric ($\vec{E}_{t}$) and magnetic ($\vec{H}_t$) fields are related through the impedance boundary condition as follows^39^:

1 $$\begin{aligned} \vec {E}_t(x, y) = \underline{\underline{Z}}_s(x, y) \cdot \hat{z} \times \vec{H}_t(x, y) \end{aligned}$$

where $\underline{\underline{Z}}_s$ is the tensorial impedance indicating anisotropic boundary conditions and $\hat{z}$ denotes the unit vector along the z-axis. For scalar (isotropic) impedance surfaces, the boundary condition is simplified as:

2 $$\begin{aligned} \vec {E}_t(x, y) = Z_s(x, y) \hat{z} \times \vec {H}_t(x, y) \end{aligned}$$

Figure 1 shows the conceptual representation of an impedance boundary condition. For spatially modulated surfaces, dielectric slabs covered by periodic subwavelength patches can be used for the realization of impedance boundary conditions. Generally, impedance surfaces support both TM and TE surface modes. Note that for the TM (TE) mode the surface wave has transverse magnetic (electric) field components in both propagation and $\hat{z}$ directions. In^40^ it has been shown that if the surface impedance is modulated sinusoidally in the propagation direction, the power will leak from the surface at a certain angle which is proportional to the impedance periodicity. In this case, the surface impedance acts as a leaky-wave antenna. However, this structure has several characteristics such as linearity and reciprocity. These properties may be rectified by adding temporal variations to the impedance surface. In spatio-temporally modulated surfaces, the impedance boundary condition changes simultaneously in space and time domains. In the general case, the impedance boundary condition can be expressed as follows:

3 $$\begin{aligned} Z_s(x, y; t) = \sum _n Z_n e^{-jnKs(x, y; t)} \end{aligned}$$

where *Ks* is the modulation phase and represents the local periodic variations in the impedance boundary condition. The coefficient $Z_n$ can be a complex number and determines the variation depth of surface impedance. For a monochromatic modulation, equation (3) is simplified as:

4 $$\begin{aligned} Z_s(x, y; t) = Z_0 + Z_{-1} e^{jKs(x, y; t)} + Z_{+1} e^{-jKs(x, y; t)} \end{aligned}$$

In this case we may define the modulation frequencies in space and time domains, which are indeed the spatial and temporal derivatives of *Ks*(*x*, *y*; *t*), respectively:

5 $$\begin{aligned} \vec {\beta }_p= & {} \nabla _{x, y} Ks(x, y; t) = \frac{\partial }{\partial x} Ks(x, y; t) \hat{x} + \frac{\partial }{\partial y} Ks(x, y; t) \hat{y} \end{aligned}$$

6 $$\begin{aligned} \omega _p= & {} -\frac{\partial }{\partial t} Ks(x, y; t) \end{aligned}$$

**Figure 2:**
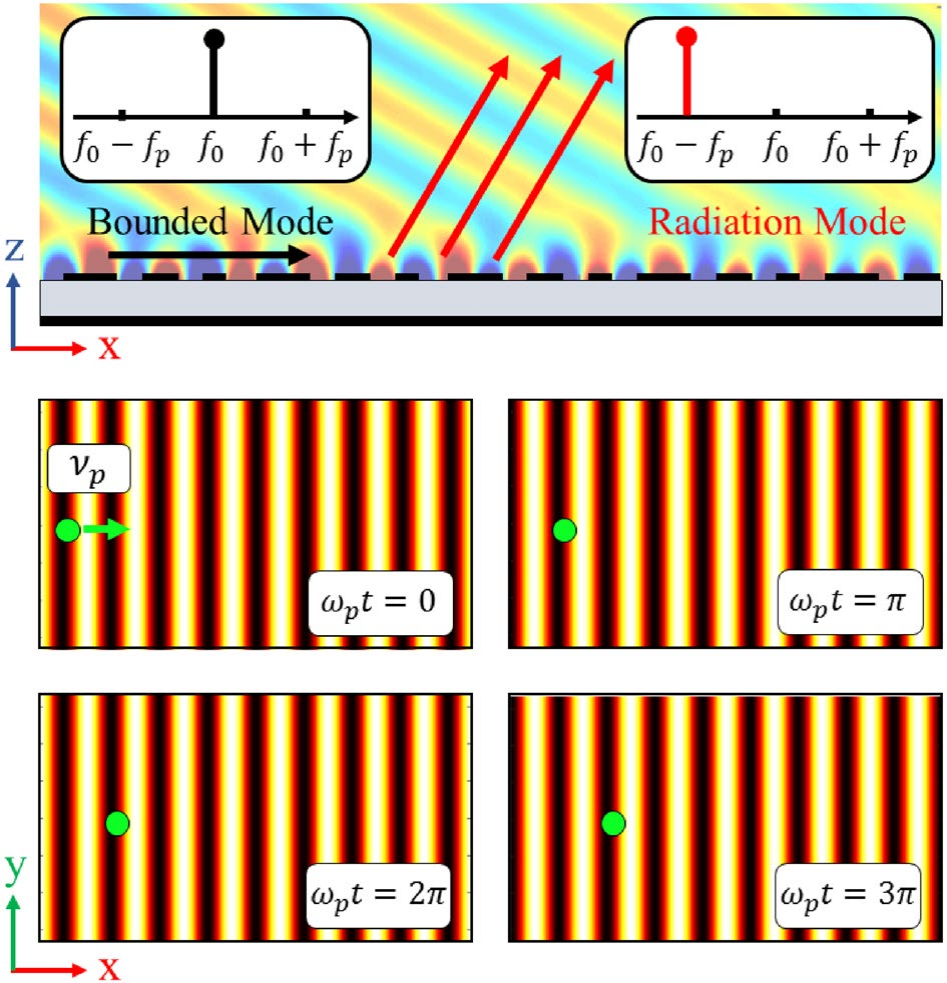
Scalar surface impedance patterns for different time slots. The circular indicator shows the progression of impedance with time.

     The frequencies $\vec {\beta }_p$ and $\omega _p$ are actually space and time domain pumping frequencies, respectively, that cause power to be coupled from surface mode to higher order Floquet modes. If the pumping frequencies are equal to zero, the power coupling will not take place and we will have a pure surface mode. Note that, *Ks* can be an arbitrary function of (x, y) in the cartesian coordinate system. In this paper, we assume that *Ks* varies only along the $\hat{x}$ direction and is constant along the $\hat{y}$ axis. Figure 2 shows an example of spatio-temporally modulated impedance surface in different time slots for $Ks(x; t) = \beta _p x - \omega _p t$ and $Z_{-1} = Z_{+1}$. In the next section, we will see that the impedance synthesized by the holographic method will be similar to that presented in Fig. 2. The progressive wave-like surface pattern travels with speed of $\nu _p = -\partial _t Ks(x) / (\nabla _x Ks(x).\hat{x}) = \omega _p / \beta _p$ along the $\hat{x}$ axis. Note that, since the boundary condition of the problem varies with time, we must use the wave equation in the time domain to analyze such structures. Thus, for the half-space above the surface the wave equation can be written as:

7 $$\begin{aligned} (\frac{\partial ^2}{\partial x^2} + \frac{\partial ^2}{\partial z^2} - \frac{1}{c^2}\frac{\partial ^2}{\partial t^2}) \psi (x, z; t) = 0 \end{aligned}$$

where c is the speed of light in vacuum. Note that, for a single frequency system without time modulation, assuming that the wave function depends on $e^{j\omega t}$, the wave equation results in the Helmholtz equation. However, in the presence of a time-varying boundary condition and the effects of higher order Floquet harmonics, the Helmholtz equation is not valid. The periodicity of boundary condition in both the time and space domains requires that the generalized Floquet-wave theory^38^ be applied. In this case, the wave function ($\psi (x, z; t)$) can be written in terms of higher order modes^38^:

8 $$\begin{aligned} \psi (x, z; t) = \sum _n \psi _n e^{j(\omega + n\omega _p)t} e^{-j(\kappa + n\beta _p)x} e^{-jk_{zn}z} \end{aligned}$$

where $\omega$ and $\kappa$ are the fundamental mode frequencies in time and space domains, respectively. The coefficient $\psi _n$ indicates the harmonic amplitude and $jk_{zn}$ is the propagation constant of n-indexed harmonic in the $\hat{z}$ direction. Using (7), $k_{zn}$ can be determined as follows:

9 $$\begin{aligned} k_{zn} = \sqrt{\frac{(\omega + n\omega _p)^2}{c^2} - (\kappa + n \beta _p)^2} \end{aligned}$$

In Eq. (8) $\omega _p$ and $\beta _p$ can be considered as degrees of freedom, of which the values may be determined by the designer. To estimate $\beta _p$, we can use holographic theory for spatially modulated surfaces.

## Holographic technique for leaky-wave metasurfaces

An effective approach for the design and synthesis of leaky-wave modulated metasurface antennas is the holographic technique, which was first proposed by Sievenpiper et al.^28,29^. In this technique, for a hologram, the required distribution of surface impedance to generate an object wave with desired direction (called $\psi _{obj}$) can be obtained as:

10 $$\begin{aligned} Z_s(x, y) = jX_0[1 + M \times \Re \{\psi _{ref}^*(x, y) \psi _{obj}(x, y)\}] \end{aligned}$$

where $\psi _{ref}$ is the reference wave excited by the electromagnetic source and asterisk ($*$) denotes the complex conjugate operation. This gradient distribution of surface impedance plays the role of holographic interferogram building up the object wave as the reference wave excites it. In Eq. (10), $X_0$ and M are average surface reactance and modulation depth, respectively. For leaky-wave holograms the modulated depth must be selected small enough (M < 0.6) in order to achieve the specified directive radiations^41^. If the radiated wave is supposed to be directed in the $\theta = \theta _0$ zenith angle, the corresponding wave (object wave) can be represented by:

11 $$\begin{aligned} \psi _{obj}(x) = e^{-jk_d \sin \theta _0 x} \end{aligned}$$

where $k_d$ indicates the wave-number at the design frequency $f = f_d$. For a planar wave-front propagating along the $\hat{x}$ axis, the reference wave can be defined as^41^:

12 $$\begin{aligned} \psi _{ref}(x) = e^{-jk_d\sqrt{1 + (\frac{X_0}{\eta _0})^2}x} \end{aligned}$$

where $\eta _0 = 120 \pi$ is the impedance of free space. Substituting (11) and (12) in (10) yields

13 $$\begin{aligned} Z_s(x) = jX_0 [1 + M\times \cos (k_d (\sqrt{1 + (\frac{X_0}{\eta _0})^2} - \sin \theta _0)x )] \end{aligned}$$

Considering the modulation phase and comparing it with the impedance distribution in the previous section, we can conclude that:

14 $$\begin{aligned} Ks(x)= & {} k_d (\sqrt{1 + (\frac{X_0}{\eta _0})^2} - \sin \theta _0) x \end{aligned}$$

15 $$\begin{aligned} Z_0= & {} jX_0, \quad Z_{+1} = Z_{-1} = j\frac{X_0 M}{2} \end{aligned}$$

16 $$\begin{aligned} \beta _p= & {} k_d (\sqrt{1 + (\frac{X_0}{\eta _0})^2} - \sin \theta _0) \end{aligned}$$

      Time dependence can be applied to the modulation phase in different ways. However, for simplicity, we assume that the temporal variation of the modulation is linear. Therefore, for dynamic structures, *Ks*(*x*; *t*) may be redefined as follows:

17 $$\begin{aligned} Ks(x; t) = k_d (\sqrt{1 + (\frac{X_0}{\eta _0})^2} - \sin \theta _0) x - \omega _p t \end{aligned}$$

## Calculation of propagation constant for asymptotic case

For the surface wave excited by the launcher in the $TM_0$ mode, the x and z components of electric field ($E_x$ and $E_z$) and the y component of magnetic field ($H_y$) may contribute to the propagation along the impedance boundary condition. Using the generalized Floquet-wave expansion we have:

18 $$\begin{aligned} E_x(x, z; t)= & {} \sum _n E_n e^{j(\omega + n\omega _p)t} e^{-j(\kappa + n\beta _p)x} e^{-jk_{zn}z} \end{aligned}$$

19 $$\begin{aligned} H_y(x, z; t)= & {} \sum _n H_n e^{j(\omega + n\omega _p)t} e^{-j(\kappa + n\beta _p)x} e^{-jk_{zn}z} \end{aligned}$$

where $\kappa$, $E_n$ and $H_n$ are unknown parameters to determined. The n-indexed Floquet mode frequencies may also be defined as follows:

20 $$\begin{aligned} \beta ^{(n)}= & {} \kappa + n\beta _p \end{aligned}$$

21 $$\begin{aligned} \omega ^{(n)}= & {} \omega + n\omega _p \end{aligned}$$

The Eigenmode analysis is an appropriate method to calculate the unknowns ($\kappa$, $E_n$ and $H_n$). Combining the impedance boundary condition and Maxwell equations for the tangential components yields:

22 $$\begin{aligned} H_{n+1} + D_n H_n + H_{n-1} = 0 \quad n = 0, \pm 1, \pm 2, \ldots \end{aligned}$$

where:

23 $$\begin{aligned} D_n = \frac{2}{M}[1 - \frac{jk_{zn}}{X_0(\omega + n \omega _p)\varepsilon _0}] \end{aligned}$$

Equation (22) relates the magnitude of n-indexed harmonic to (n+1) and (n-1)-indexed modes, which form a set of infinite equations with infinite unknowns. These equations resemble the dispersion relations in the pure-space modulated case^40^, except that $\omega ^{(n)} = \omega + n\omega _p$ is used instead of $\omega$. In order to solve this system of equations, we must truncate the number of modes for analysis.

For the asymptotic case, if the modulation index tends to zero ($M\rightarrow 0$), the numerator of fraction in equation (23) needs to be zero to achieve nontrivial solutions. In this case we have:

24 $$\begin{aligned} 1 - \frac{j\sqrt{\frac{(\omega + n\omega _p)^2}{c^2} - (\kappa + n\beta _p)^2}}{X_0(\omega + n \omega _p) \varepsilon _0} = 0 \end{aligned}$$

Therefore, the set of asymptotic curves for $\kappa$ can be obtained from the following equation:

25 $$\begin{aligned} \kappa = -nk_d (\sqrt{1 + (\frac{X_0}{\eta _0})^2} - \sin \theta _0) \pm \frac{\omega + n\omega _p}{c} \sqrt{1 + (\frac{X_0}{\eta _0})^2} \quad n = 0, \pm 1, \pm 2, \ldots \end{aligned}$$

**Figure 3:**
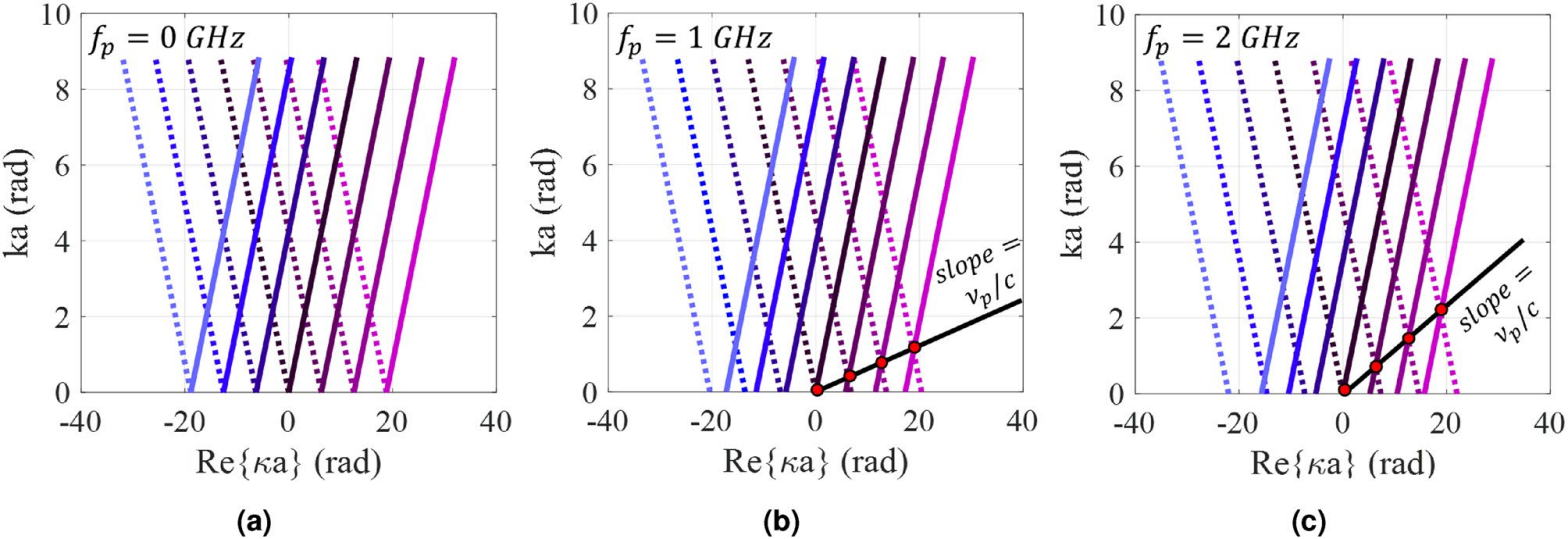
Dispersion curves for different pumping frequencies. (**a**) $f_p = 0$, (**b**) $f_p = 1$ GHz, and (**c**) $f_p = 2$ GHz.

Figure 3 shows the asymptotic dispersion curves for the cases of $f_p = 0$, $f_p = 1$ GHz and $f_p = 2$ GHz. The solid and dashed curves represent forward and backward solutions, respectively. These curves can be used as initial guesses for the calculation of eigenstates of the modulated case. Observe that, injection of temporal variations into the modulated impedance function imposes some asymmetry on the dispersion curves, leading to a nonreciprocal response. As shown in Fig. 3, in the presence of temporal modulation, the Brillouin diagram is parallel to the line with slope of $\nu _p / c$ and the period of Brillouin zone (the distance between adjacent red points in Fig. 3) is $\sqrt{\beta _p^2 + (\omega _p/c)^2}$. Observe that increasing the temporal pumping frequency results in the increase of the degree of asymmetry. This property will be described in more detail in the following section.

## Determination of propagation constant in the presence of space-time modulation

As discussed in the previous section, The infinite system of equations in (22) should be solved for the determination of the exact values of eigenstates in the presence of space-time modulation, which is achieved by safely truncating the number of modes without generating a significant error. The following nonlinear equation is obtained by some mathematical simplifications^38,40^:

26 $$\begin{aligned} D_n - \frac{1|}{D_{n+1}} - \frac{1|}{|D_{n+2}} - \frac{1|}{|D_{n+3}} - \cdots \frac{1|}{D_{n-1}} - \frac{1|}{|D_{n-2}} - \frac{1|}{|D_{n-3}} - \cdots = 0 \end{aligned}$$

**Figure 4:**
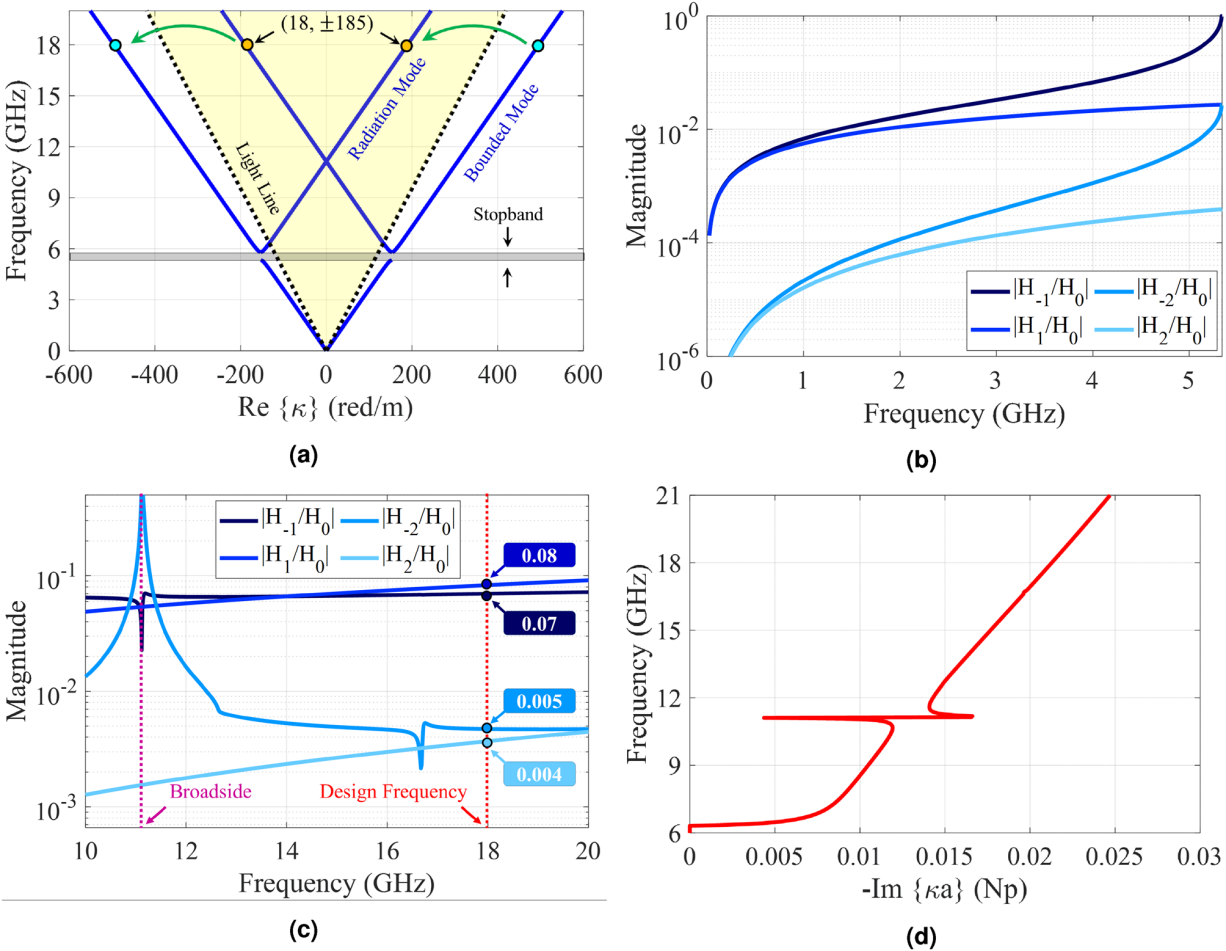
Analytical results of the proposed hologram in the pure-space modulation mode ($f_p = 0$ GHz) for the case of $X_0 = 0.85\eta _0$ and $M = 0.2$. (**a**) Dispersion diagram. (**b**) Normalized magnitudes of different harmonics as a function of *ka* in the lower band. (**c**) Normalized magnitudes of different harmonics in the leaky-wave region. (**d**) Attenuation constant curve as a function of frequency.

In order to arrive at sufficiently accurate solutions, we consider 21 Floquet modes (namely $n = 0, \pm 1, \pm 2, \ldots ,\pm 10$). The solutions of Eq. (26) determine the complex values of wave-number at a given frequency. Figure 4a shows the dispersion diagram of hologram for the case of $f_p = 0$ GHz. The design frequency is 18 GHz, and the beam angle at 18 GHz is set at $\theta _0 = 30^\circ$. The average impedance ($X_0$) and modulation index (M) are selected as $0.85\eta _0$ and 0.2. Therefore, the corresponding impedance may be obtained as:

27 $$\begin{aligned} Z_s(x) = j 0.85 \eta _0 [1 + 0.2 \cos (377.25(\sqrt{1 + 0.85^2} - 0.5)x)] = j320.44[1 + 0.2\cos (306.49 x)] \end{aligned}$$

Figure 4b shows the normalized magnitudes of the space harmonics in the lower band. Note that the first stop band occurs at $ka = 2.3$ ($f \approx 5.3\, GHz$). In this region all the space harmonics are slow waves and their corresponding propagation wavenumbers are real. Observe that, around the first stop band, the following relationships can be established among the magnitudes of space harmonics:

28 $$\begin{aligned} |H_{-1}| = |H_0|, \quad |H_{-2}| = |H_1|, \quad \cdots \end{aligned}$$

**Figure 5:**
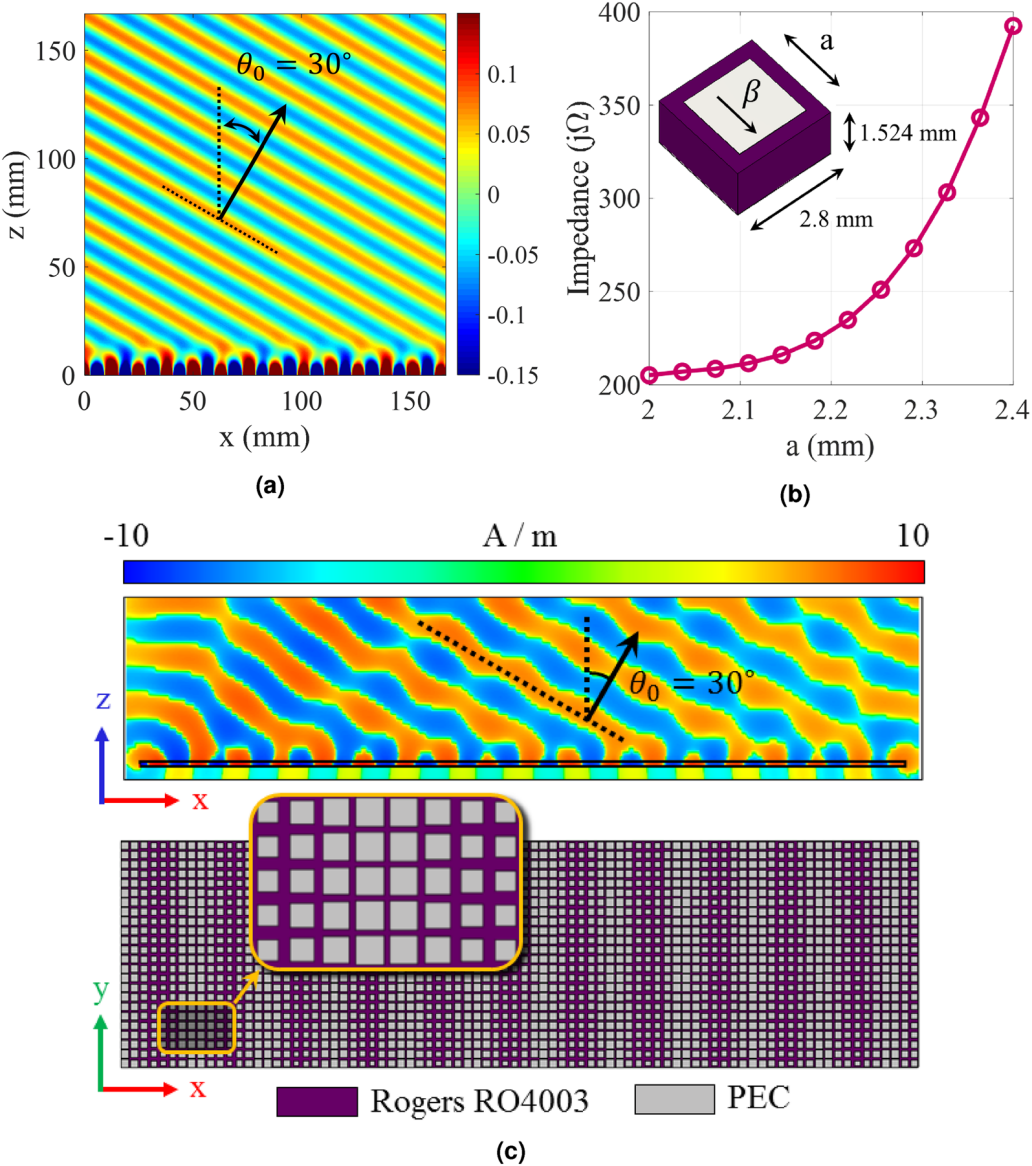
(**a**) Analytical results for total magnetic field at 18 GHz. (**b**) Proposed unit-cell and its equivalent surface impedance as a function of patch width. (**c**) Static hologram implemented by an quasi-periodic array of printed square patches.

**Figure 6:**
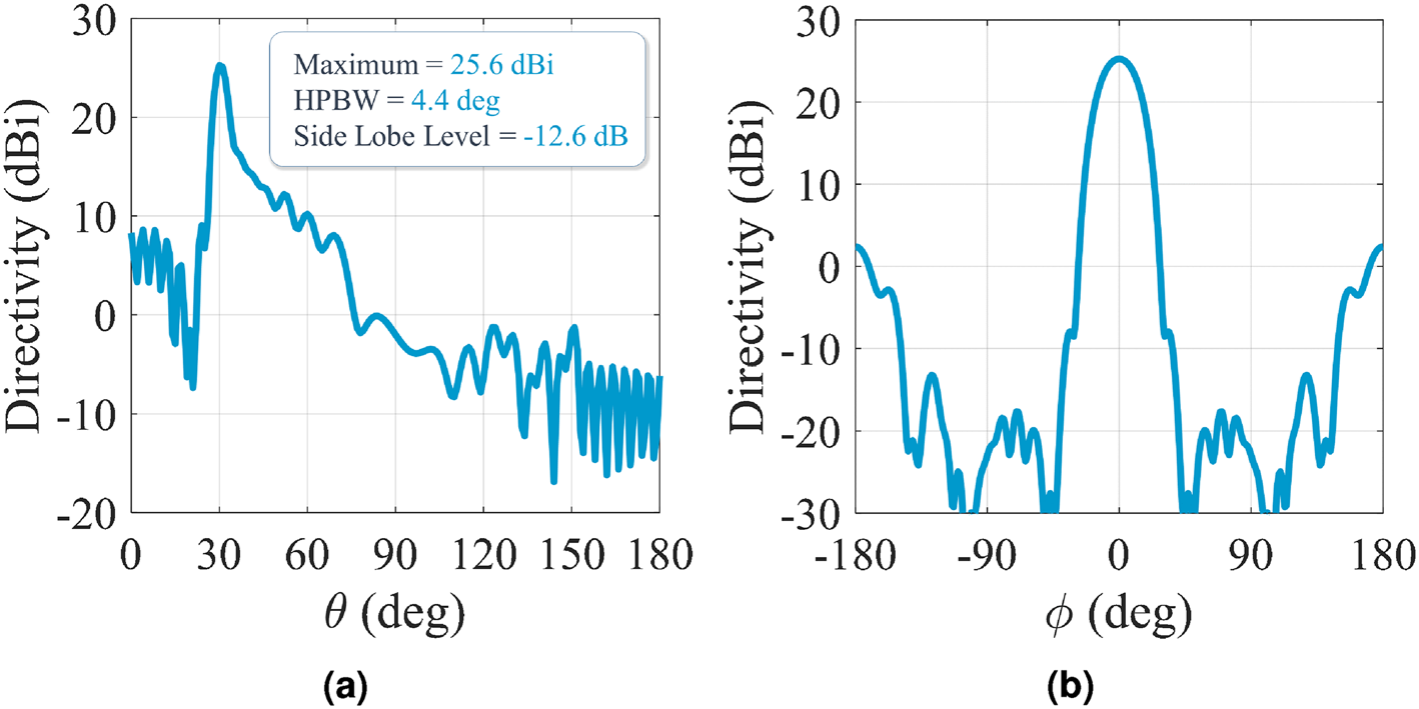
Simulated radiation patterns of the proposed hologram in Fig. 5 at: (**a**) $\phi = 0^\circ$ and (**b**) $\theta = 30^\circ$ planes. The operation frequency is 18 GHz.

As the frequency increases, the wave leaks gradually from the surface and then $\kappa$ becomes complex. In Fig. 4c the magnitudes of space harmonics in the leaky-mode region are plotted. Observe that, except in a limited region (around $ka = 4.7$ which occurs at 11.1 GHz), the mode numbers $n = \pm 1$ are predominant. Note also that, in the vicinity of $f = 11.1$ GHz the transition region occurs from the backward to forward modes. This region is known as the open-stopband region where the antenna gain may be drastically reduced. The sudden drop in the radiation mode (n = − 1) can be observed in Fig. 4c. The attenuation constant curve is depicted in Fig. 4d. The open-stopband effect on the attenuation can also be seen around 11.1 GHz. The total magnetic field at 18 GHz is also plotted in Fig. 5a, which show that, in the transmission mode, the coupling occurs from the surface to radiation mode with the radiation angle of 30 degrees. Also, in the reception mode, the maximum coupling occurs at the incoming angle of 30 degrees, which confirms the accuracy of the design. To confirm the accuracy of the analytical results, a one-dimensional hologram is designed by exploiting the subwavelength quasi-periodic patches, as shown in Figs. 5b and 5c. The dimensions of the antenna are selected equal to $14\lambda \times 4 \lambda$ ($\lambda$ as the free-space wavelength), so that the surface wave has enough space for effective radiation. Rogers RO4003 (with $\varepsilon _r = 3.55$, $\tan \delta = 0.0027$, and $h = 1.524$ mm) is chosen as the dielectric substrate. Results in Fig. 5b show that the realizable impedance range of this unit-cell is j205 to j392 $\Omega$ which covers the impedance variations in Eq. (27). Figure 5c shows the simulated magnetic field in the vicinity of the hologram surface. The simulation is performed by CST Microwave Studio package with finite integration technique (FIT) method^42^. Figure 6 presents the far-zone radiation patterns for $\phi = 0^\circ$ and $\theta = 30^\circ$. The maximum directivity is 25.6 dBi at 18 GHz and its side lobe level is better than − 12.5 dB.

**Figure 7:**
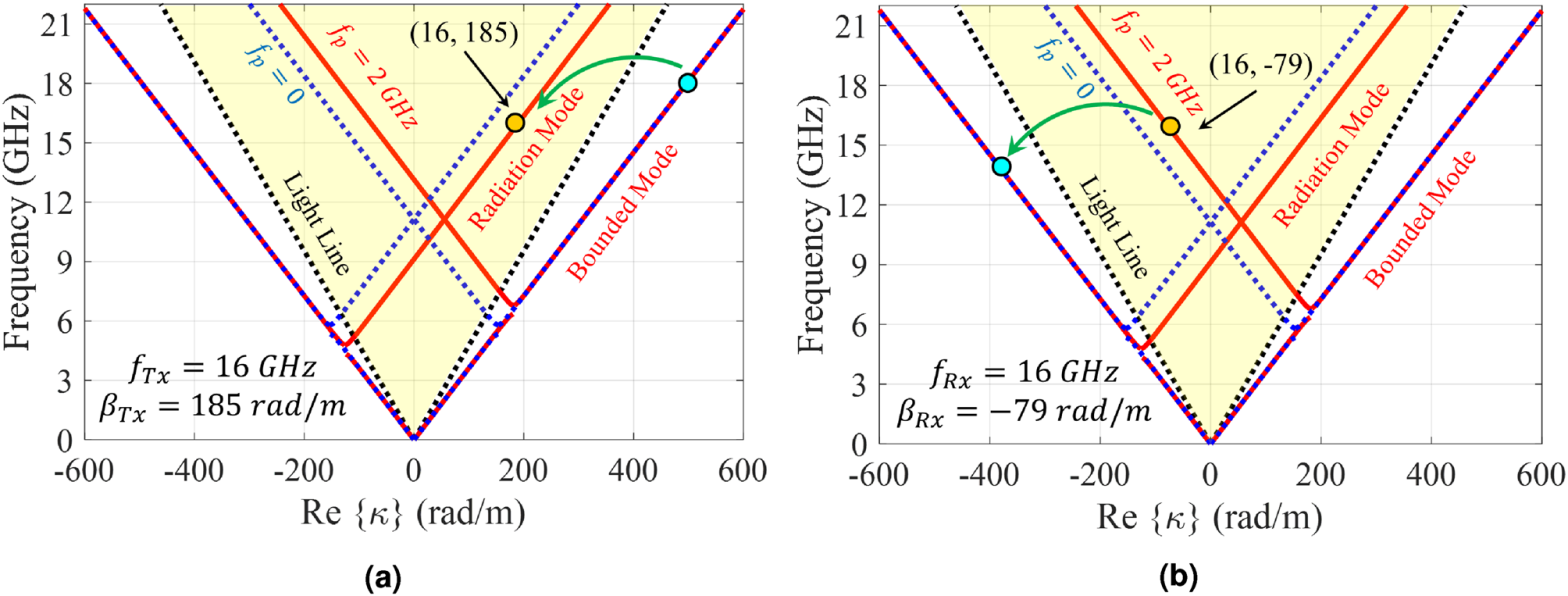
Dispersion diagram of the proposed spatio-temporally modulated hologram for the case of $f_p = 2$ GHz in (**a**) transmission mode, and (**b**) receiving mode.

Injecting temporal modulation into the designed hologram, imposes some modifications to the dispersion diagram. Figure 7 shows the dispersion curves for the case of $f_p = 2$ GHz. Its impedance distribution has the following form:

29 $$\begin{aligned} Z_s(x; t) = j320.44[1 + 0.2 \cos (306.49x - 4\pi \times 10^9 t)] \end{aligned}$$

**Figure 8:**
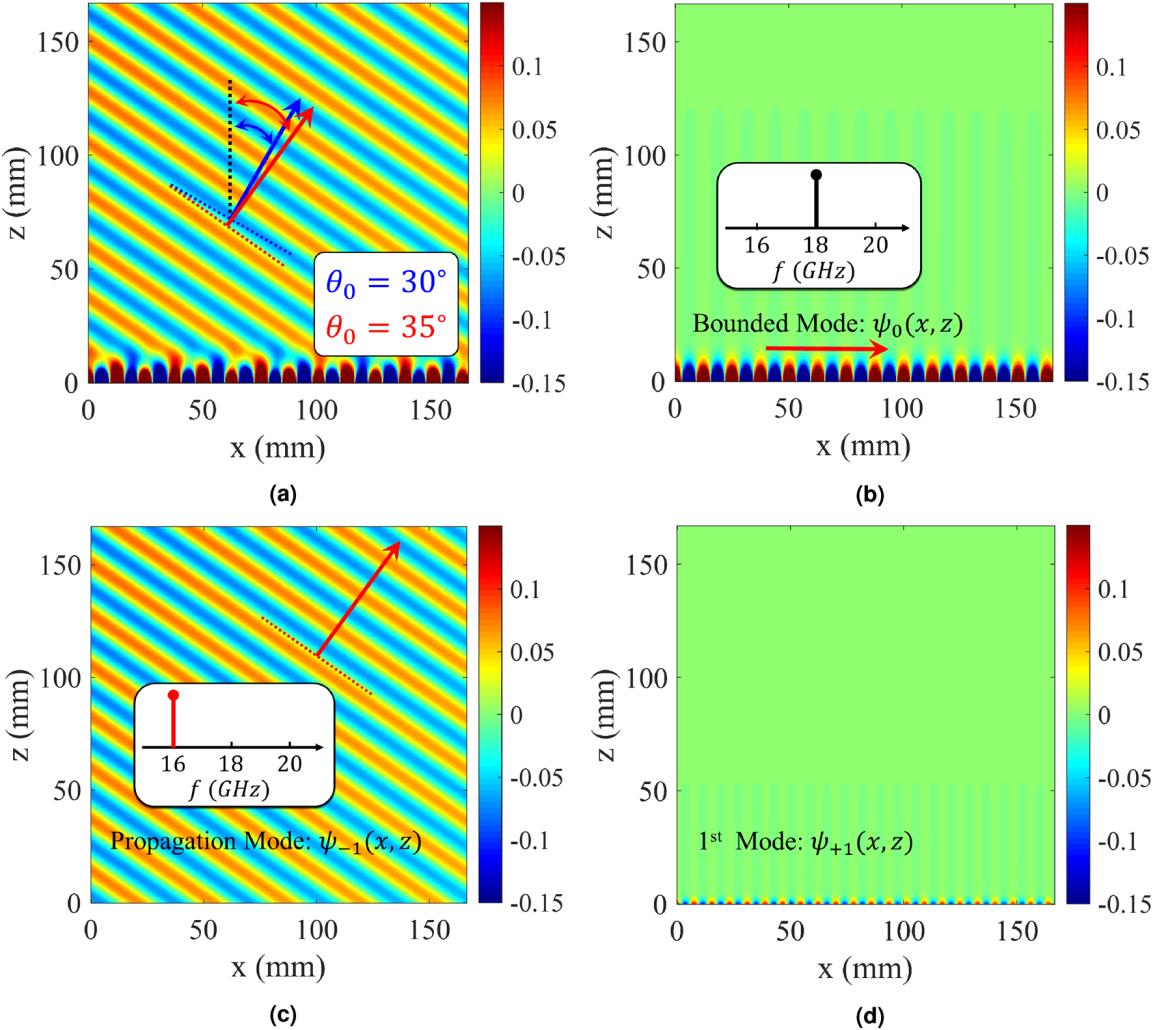
Analytical results of the proposed spationtemporally modulated hologram in transmission mode. (**a**) Total field, (**b**) fundamental mode, (**c**) radiation mode, and (**d**) (+1)-index mode.

**Figure 9:**
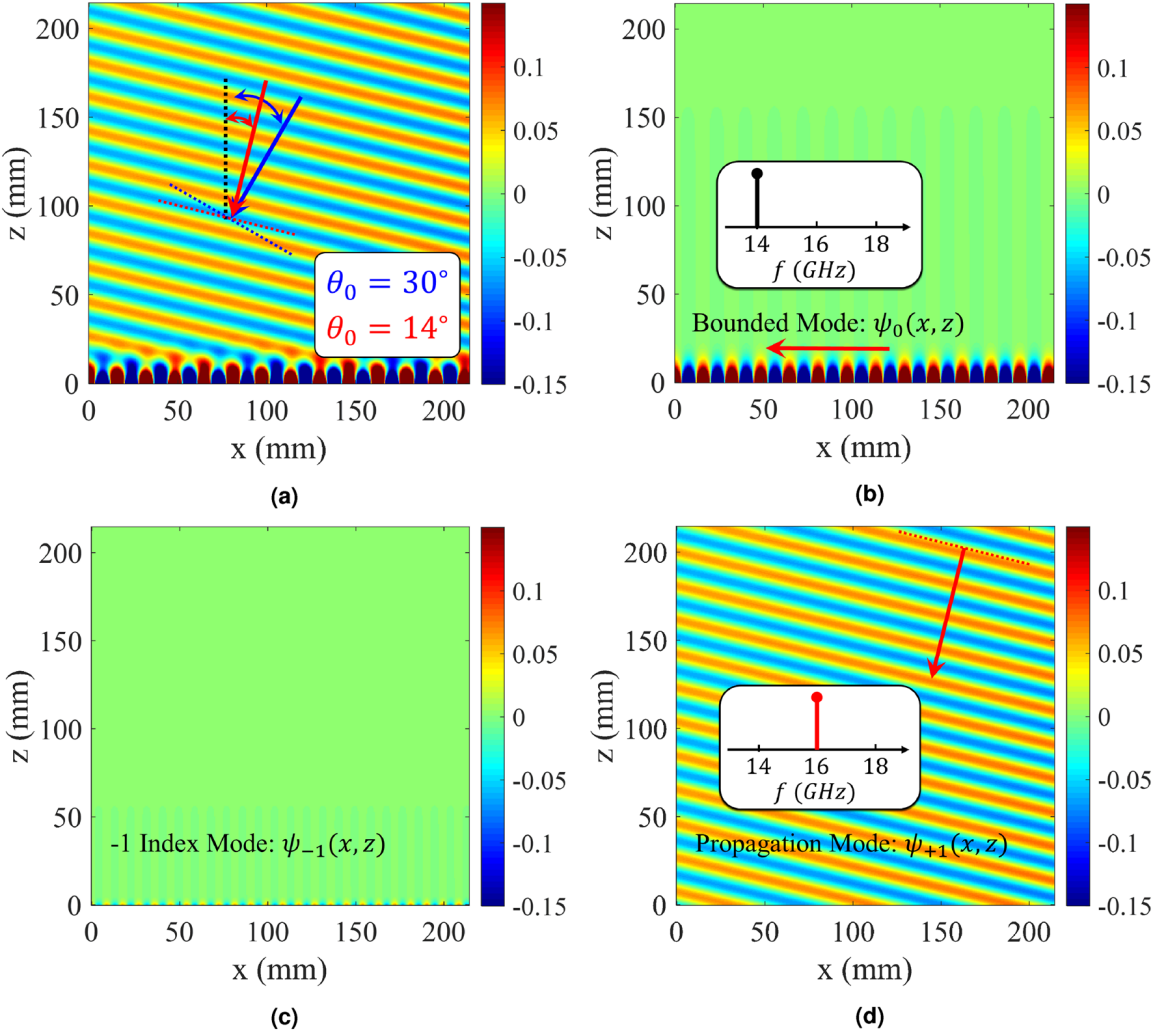
Analytical results of the proposed spationtemporally modulated hologram in receiving mode. (**a**) Total field, (**b**) fundamental mode, (**c**) (− 1)-index mode, and (**d**) radiation mode.

**Figure 10:**
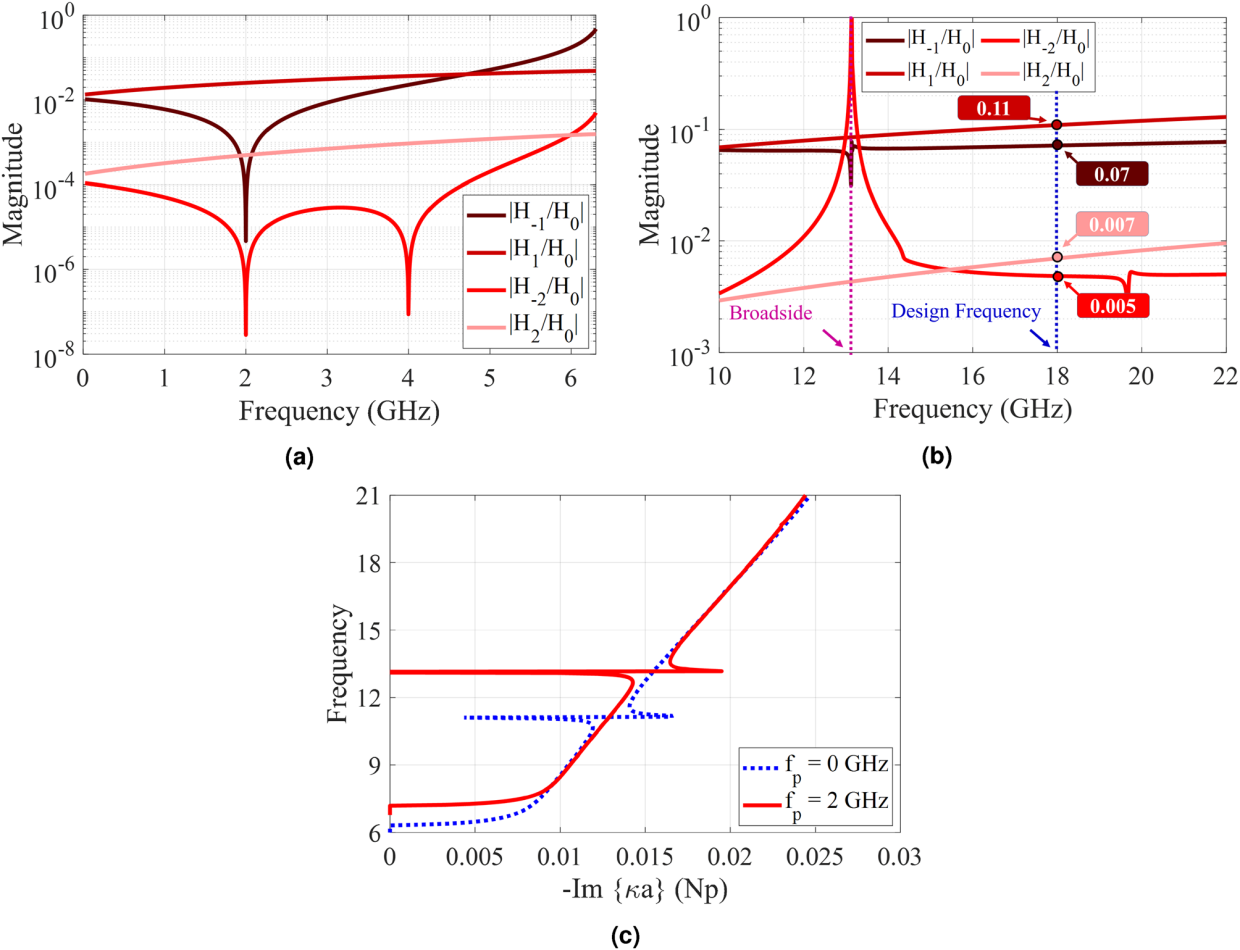
Analytical results of the proposed spatio-temporally modulated hologram for the case of $X_0 = 0.85\eta _0$, $M = 0.2$ and $f_p = 2\, {\text{GHz}}$. (**a**) Normalized magnitudes of different harmonics as a function of frequency in the lower band. (**c**) Normalized magnitudes of different harmonics in the leaky-wave region. (**d**) Attenuation constant curve as a function of frequency.

Observe that, in the presence of temporal modulation, the curves corresponding to negative and positive harmonics move down and up respectively. This displacement is proportional to the pumping frequency ($f_p$). As the pumping frequency increases, the dispersion curve for $n = -1$ tends towards the light-line, indicating that the radiation mode tends towards the end-fire direction. Also, for $n = +1$, the radiation curve gets closer to the vertical axis. To investigate nonreciprocity, we first consider the antenna operation in transmission mode. Suppose that the excitation frequency of the antenna is 18 GHz, which operates at the fundamental mode (surface wave). According to the dispersion diagram in Fig. 7a, its corresponding phase constant is equal to $\beta ^{(0)} = 492$ (rad/m). Furthermore, using (20) the phase constant for radiation mode ((-1)-indexed mode) can be obtained as $\beta ^{(-1) } = \beta ^{(0)} - \beta _p \approx 185$ (rad/m). Observe in the dispersion diagram that the corresponding frequency at this phase constant is 16 GHz (namely $f^{(-1)} = f^{(0)} - f_p$), which shows the Doppler-shift effect in the proposed antenna. According to the calculated phase constant, the beam orientation is directed at 35 degrees. In Fig. 8 the analytical results of total magnetic field and Floquet-modes of $n = 0, \pm 1$ are plotted separately. Figure 8 indicates that a strong coupling can be observed between 0-indexed and (− 1)-indexed Floquet modes. In this case, most of the power is divided into the fundamental and (− 1)-indexed modes, but the (+ 1)-indexed mode has a small portion of the power. The radiation mode is oriented in the direction of 35 degrees. The blue and red arrows in Fig. 8a indicate the beam directions for pure-space and space-time modulated cases, respectively. When the antenna is in the reception mode, for the 16 GHz incoming wave, the corresponding phase constant is entirely different from that of the transmission case. Using (21), for (+ 1)-indexed mode with frequency of 16 GHz, the 0-indexed mode propagates at $f^{(0)} = f^{(+1)} - f_p = 14$ GHz. In this case, the phase constant of (+1)-indexed mode is equal to $\beta ^{(+1)} \approx -79$ (rad/m) and the angle at which the maximum power is coupled to the surface is 14 degrees (as shown in Fig. 9). Note that the surface is excited at 14 GHz instead of 16 GHz. Figure 10a shows the magnitudes of the space harmonics as a function of the excitation frequency. In this case, the first stopband edge occurs at 6.4 GHz. Observe that, due to the temporal injection, the curves with negative (positive) mode numbers are shifted toward higher (lower) frequencies. Therefore, due to these displacements, the magnitudes of $|H_{-2}|$ and $|H_1|$ at the stopband edge will no longer be the same. Figure 10b gives the amplitude curves at higher frequencies at which the metasurface operates in the leaky mode. Similar to the pure-space modulated structure, the mode numbers of $n = \pm 1$ have the dominant magnitudes at the design frequency. Note that, in this case, the broadside radiation occurs at the excitation frequency of *f* = 13.1 GHz. The sudden change in attenuation constant due to the open stopband effect can also be seen in Fig. 10c.

To summarize our analysis we can conclude that, for the antenna in the transmission mode, the maximum coupling between the surface and radiation modes occurs at the angle of 35 degrees, whereas for the reception case the radiation and surface modes have the maximum coupling at the incoming angle of 14 degrees . Therefore, the antenna does not behave the same in the transmission and reception modes, which confirms its nonreciprocal behavior.

## Beam scanning properties

**Figure 11:**
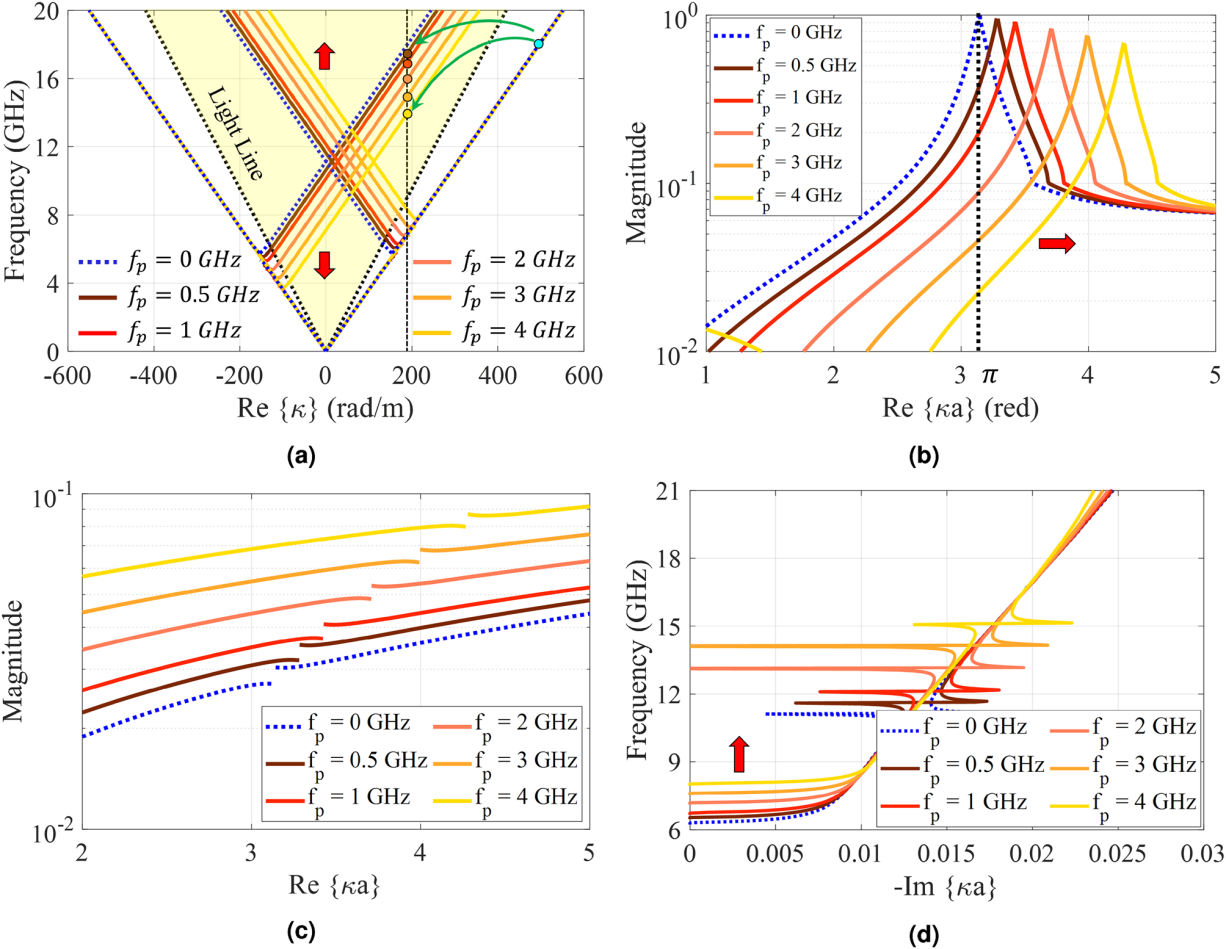
Analytical results of the proposed hologram for different values of $f_p$. (**a**) Dispersion diagram. (**b**) Normalized magnitude of higher (-1)-indexed Floquet modes ($|H_{-1}/H_0|$) versus $\kappa a$. (**c**) Normalized magnitude of higher (+1)-indexed Floquet modes ($|H_{+1}/H_0|$) versus $\kappa a$. (**d**) Attenuation curves.

Leaky-wave holograms can be suitable choices for automotive sensors, coherent tomography, real-time spectrum analysis and tracking applications owing to their beam scanning capabilities, which can be controlled by source frequency. Referring to the dispersion diagram in Fig. 4a, for a pure-space modulated hologram, the corresponding phase constant increases by enhancing excitation frequency. This means that, the radiation beam tends towards the end-fire direction at higher frequencies. In temporally modulated holograms, beam scanning can be achieved through another mechanism. In this case, the beam direction varies by changing the pumping frequency ($f_p$) without the need to change the source frequency. The calculated dispersion diagrams for different values of $f_p$ are plotted in Fig. 11a. Observe that for a given phase constant, the (− 1)-indexed curve tends towards the light-line as the pumping frequency increases. The dispersion curve for (+1)-indexed mode moves in opposite direction indicating that the incoming wave angle for reception case tends towards the broadside direction. It is worth noting that, this movement changes the radiation frequency in the forward mode, and consequently the radiation beam scans toward the end-fire direction. The spot progression in Fig. 11a for $\beta ^{(-1)} \approx 185$ (rad/m) indicates this phenomenon. The space harmonics with mode numbers of $n = -1$ and $n = +1$ in the first triangle are given in Figs 11b and 11c, respectively. For a given value of $\kappa a$, Increasing the temporal modulation frequency, decreases (increases) the magnitude of negative (positive) modes. Fig. 11d compares the attenuation constants for different values of $f_p$. The open stopband region shifts toward higher frequencies as the temporal frequency increases.

**Figure 12:**
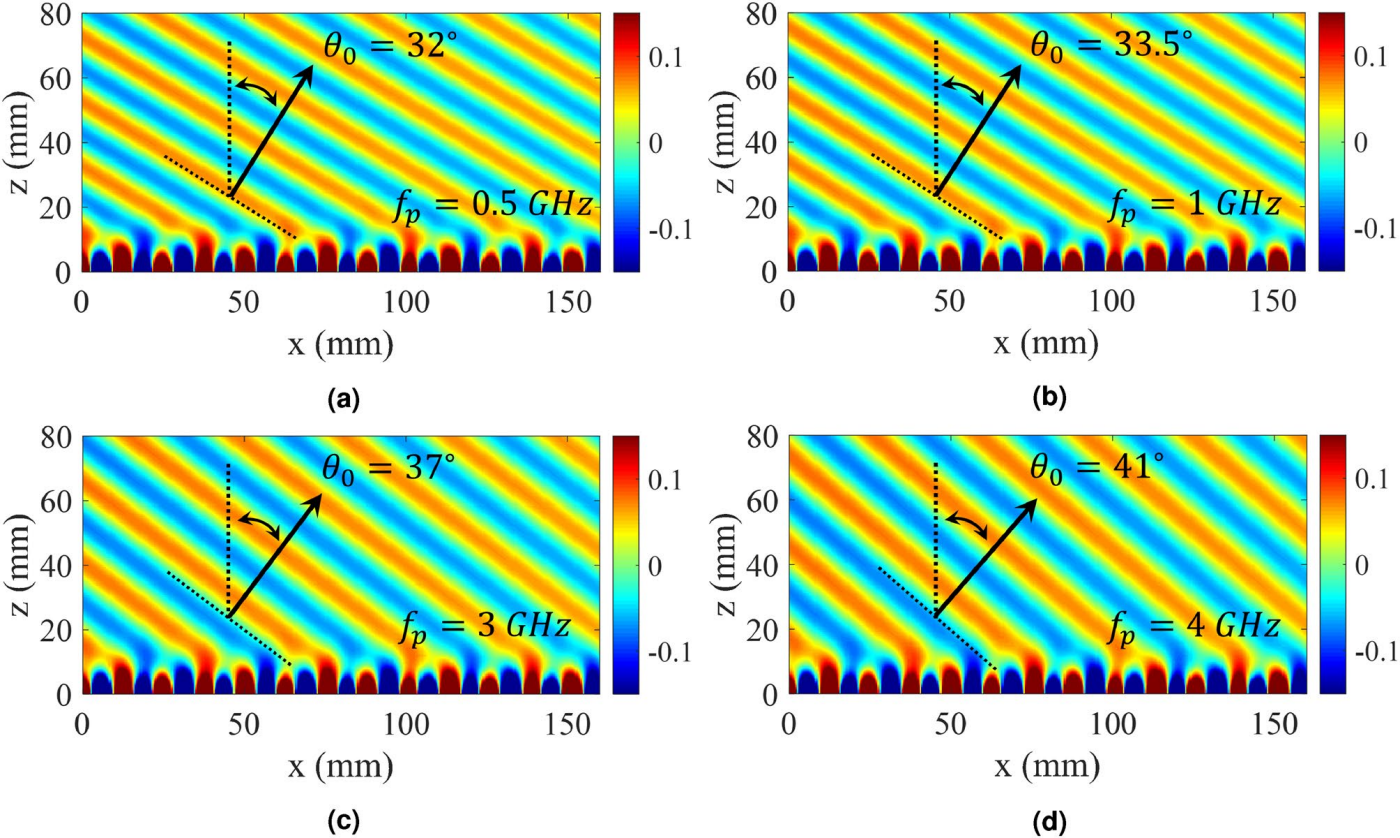
Total fields for different values of pumping frequencies. (**a**) $f_p = 0.5 \, {\text{GHz}}$, (**b**) $f_p = 1 \, {\text{GHz}}$, (**c**) $f_p = 3 \, {\text{GHz}}$, and (**d**) $f_p = 4 \, {\text{GHz}}$.

Figure 12 shows the calculated fields for different values of $f_p$. Results show that the beam direction changes from $\theta = 30^\circ$ to $\theta = 41^\circ$ with pumping frequency increasing from 0 to 4 GHz. It should be noted that the source frequency is fixed at 18 GHz.

## Conclusion

In this paper, the generalized Floquet-wave expansion method is utilized to accurately calculate the propagation characteristics of temporally modulated holograms. The combination of holographic technique as a synthesis method and Floquet-wave expansion can form a fully-analytical model for implementation of leaky-wave metasurfaces. It has been shown that temporally modulated holograms can be effectively used as nonreciprocal antennas. This nonreciprocity can be obtained by properly displacing the dispersion curve so that the beam is directed at the desired spherical angle. Owing to their embedded feeding networks, leaky-wave antennas can be appropriate alternatives to nonreciprocal metareflectors and transmitarrays as radiators of integrated transceivers.
